# Personalized treatment of advanced non-small-cell lung cancer in routine clinical practice

**DOI:** 10.1007/s10555-016-9612-6

**Published:** 2016-03-12

**Authors:** Robert Pirker, Martin Filipits

**Affiliations:** Department of Medicine I, Medical University of Vienna, Währinger Gürtel 18-20, 1090 Vienna, Austria

**Keywords:** ALK, Biomarker, Cetuximab, EGFR mutations, Liquid biopsy, Necitumumab

## Abstract

Personalized treatment of patients with advanced non-small-cell lung cancer based on clinical and molecular tumor features has entered clinical routine practice. The 2015 pathological classification of lung cancer mandates immunohistochemical and molecular analysis. Therapeutic strategies focused on inhibition of angiogenesis and growth factor receptor signaling. Inhibitors of angiogenesis and monoclonal antibodies directed against the epidermal growth factor receptor have shown efficacy in combination with chemotherapy. Mutations in the epidermal growth factor receptor and anaplastic lymphoma kinase have become clinically relevant therapeutic targets. Immune checkpoint inhibitors are also entering routine clinical practice. Identification of predictive biomarkers is essential and faces several challenges including tumor heterogeneity and dynamic changes of tumor features over time. Liquid biopsies may overcome some of these challenges in the future.

## Introduction

Lung cancer is the most commonly diagnosed cancer worldwide with 1.82 million cases in 2012 [1]. Pathological classification into small-cell lung cancer (SCLC) and non-small-cell lung cancer (NSCLC) has been standard for assessment of prognosis and selection of treatment for many years. NSCLC comprises about 80 % of all lung cancers. Within the last decade, however, further subclassification of NSCLC into non-squamous-cell and squamous-cell NSCLC entered clinical practice. These changes became necessary after specific anticancer drugs had been shown to have efficacy or toxicity in certain subtypes of NSCLC [2, 3]. Molecular classification also entered routine practice, mainly through the identification of molecular therapeutic targets. The 2015 classification of lung cancer now mandates immunohistochemical and molecular analysis in routine clinical practice [4].

Lung cancer is among those cancers with the highest mutation rates [5]. Genome-wide sequencing revealed specific changes in tumors [6–11]. Among adenocarcinomas, frequent genetic alterations are mutations in p53, Kirsten rat sarcoma viral oncogene homolog (*KRAS*), cyclin-dependent kinase inhibitor 2A (*CDKN2A*), mixed-lineage leukemia 3 (*MLL3*), serine/threonine kinase 11 (*STK11*), and the epidermal growth factor receptor (*EGFR*) [6–9]. Among squamous-cell NSCLC, frequent mutations are seen in phosphatidylinositol-4-5-biphosphate 3-kinase (*PIK3CA*), sex-determining region Y-related gene family 2 (*SOX2*), *CDKN2A*, fibroblast growth factor receptor 1 (*FGFR1*), and *MLL3* [10]. Among small-cell lung cancers, frequent mutations are seen in retinoblastoma 1 (*RB1*), E1A-binding protein p300 (*EP300*), *MLL2*, and *PIK3CA* [11].

The identification of specific mutations including driver mutations in certain histological subtypes has led to molecular subclassification of NSCLC and also opened therapeutic opportunities for personalized medicine (also termed precision medicine) based on these molecular targets. Driver mutations involved in tumor growth were of particular therapeutic interest because their blockade opened the possibility for improved clinical outcome of patients. Driver mutations of current therapeutic relevance for routine practice are those seen in the epidermal growth factor receptor (EGFR), anaplastic lymphoma kinase (ALK), and ROS1. The identification of driver mutations in squamous-cell carcinomas and small-cell lung cancer may have therapeutic implications in these carcinomas in the near future [10]. Immune checkpoint inhibitors have shown efficacy in phase III trials in patients with advanced NSCLC who have been pretreated with chemotherapy [12, 13]. Research on predictive biomarkers is currently ongoing.

Here we summarize the current status of personalized treatment based on predictive biomarkers in patients with advanced NSCLC in routine clinical practice.

## Tumor histology

Classification of lung cancer into NSCLC and SCLC was sufficient for management of patients with lung cancer in routine practice for many years. During the last decade, however, NSCLC subclassification by means of immunohistochemistry and molecular factors has become clinically mandatory [4]. Reasons for this are better understanding of tumor biology, preferential efficacy or toxicity of novel drugs in subtypes of NSCLC, and the demonstration of therapeutically relevant driver mutations in subsets of NSCLC.

The interaction between tumor histology and drug efficacy was observed in case of pemetrexed which was shown to have preferential efficacy in patients with non-squamous NSCLC. In a phase III trial in chemo-naïve patients with advanced NSCLC, cisplatin plus pemetrexed increased overall survival compared to cisplatin plus gemcitabine in patients with non-squamous-cell NSCLC [2]. Bevacizumab was mainly developed in only patients with non-squamous NSCLC because an early clinical trial suggested increased rates of bleedings in patients with squamous-cell NSCLC [3]. Based on these findings, subtyping of NSCLC into non-squamous-cell and squamous-cell NSCLC entered routine clinical practice.

The subclassification of NSCLC has also become necessary for guiding molecular analyses because EGFR mutations and other driver mutations were preferentially detected in patients with adenocarcinomas. Patients with advanced adenocarcinomas are currently tested for EGFR mutations, ALK aberrations, and ROS1 in routine clinical practice. Based on local practice and possibilities, additional molecular markers are determined. The 2015 classification of lung cancer requires immunohistochemical and molecular analyses of tumors [4].

## Customized chemotherapy

Customized chemotherapy based on molecular tumor features has extensively been studied and is beyond the present review. Research has focused on ERCC1, RRM1, and thymidylate synthase as potential biomarkers. ERCC1 was initially shown to predict outcome of adjuvant chemotherapy [14]. The validation in the LACE Bio project, however, failed to confirm these findings [15]. Palliative chemotherapy based on ERCC1 levels failed to demonstrate a survival advantage compared to a standard protocol [16]. Because no biomarker could reliably predict clinical outcome of chemotherapy, customized chemotherapy based on molecular markers has not entered routine clinical practice.

## EGFR tyrosine kinase inhibitors

Blockade of EGFR by tyrosine kinase inhibitors (TKIs) or monoclonal antibodies improved outcome in patients with advanced NSCLC (for review, see [17–19]).

First-generation EGFR TKIs, e.g., gefitinib and erlotinib, have initially been evaluated in unselected patients with advanced NSCLC who had progressed during or after palliative chemotherapy (for review, see [17, 18]). In 2004, EGFR-activating mutations have been identified in tumors of those patients who had shown excellent responses to EGFR TKIs [20–22]. EGFR mutations in advanced adenocarcinomas are detected in approximately 10–15 % of Caucasian patients and 40–60 % of patients of Southeast Asian ethnicity.

The identification of EGFR mutations led to phase III trials comparing EGFR TKIs with palliative chemotherapy in patients with advanced EGFR mutation-positive NSCLC (for review, see [17, 18]). EGFR TKIs (afatinib, erlotinib, gefitinib) administered until disease progression prolonged progression-free survival and improved quality of life in comparison to first-line chemotherapy in patients with EGFR-activating mutations but were inferior to chemotherapy in patients without these mutations. Afatinib also increased overall survival compared to chemotherapy, and this survival benefit was seen in patients with exon 19 deletions [23]. The results of these phase III trials led to the approval of EGFR TKIs for first-line therapy of patients with advanced EGFR mutation-positive NSCLC and to clinical practice guidelines recommending, first, EGFR mutation testing for all patients with advanced adenocarcinomas, and, second, EGFR TKIs as the preferred first-line therapy for patients with advanced EGFR mutation-positive NSCLC [24–27]. Recently, afatinib was also shown to increase survival compared to erlotinib in patients with advanced squamous-cell NSCLC who had progressed after pretreatment with chemotherapy [28].

After median progression-free survival times of approximately 9–13 months, patients with EGFR mutation-positive NSCLC usually acquire resistance to EGFR TKIs. Acquired resistance may be pharmacological or caused by molecular changes of the tumor [29, 30]. Molecular mechanisms of acquired resistance are EGFR target alterations in about 60 %, non-EGFR bypass track alterations in about 20 % of patients, histological transformation to small-cell lung cancer, epithelial–mesenchymal transformation, and yet to be identified mechanisms in the remaining patients [29, 30]. EGFR target alterations are mainly T790M mutations within exon 20 which occur alone in 40–55 % and in combination with EGFR amplification in 10 % of the patients. The T790M mutation leads to an enhanced affinity for adenosine triphosphate, thereby reducing the ability of reversible EGFR TKIs to bind to the tyrosine kinase domain of EGFR. Treatment options at the time of acquired resistance are continuation of treatment with TKIs in selected patients with clinically slow progression, addition of local radiotherapy, and switch to either chemotherapy or third-generation EGFR TKIs. Cetuximab combined with either afatinib or chemotherapy has also been studied [31, 32].

Third-generation TKIs are active against EGFR-activating mutations and the T790M resistance mutation and have only limited efficacy against wild-type EGFR. Drugs in clinical development include osimertinib, rociletinib, and HM61713 [33–37]. Osimertinib and rociletinib have shown clinical efficacy in phase I/II trials in patients with acquired resistance to first-generation or second-generation TKIs and are currently compared to standard treatment in phase III trials in patients with advanced EGFR mutation-positive NSCLC [35, 36]. Patients undergoing treatment with third-generation EGFR TKIs will eventually also develop resistance through the emergence of one of the following three resistance mutations: EGFR L718Q, L844V, and C797S [38, 39].

### EGFR mutations as predictive biomarkers

EGFR TKIs were found to be preferentially active in patients with adenocarcinomas, never-smokers, and patients of Southeast Asian ethnicity. In 2004, EGFR activating mutations have been identified in patients with good responses to TKIs [20–22]. Phase III trials then demonstrated superior outcome of TKIs compared to first-line chemotherapy in patients with advanced EGFR mutation-positive NSCLC (for review, see [17, 18]). These results led to the approval of EGFR TKIs for first-line therapy in patients with advanced EGFR mutation-positive NSCLC. These approvals then required the implementation of EGFR mutation testing in routine clinical practice for patients with adenocarcinomas.

EGFR mutation testing has now been established in routine clinical practice in most cancer centers of the world. In this context, close co-operation and communication between treating physicians and pathologists have been important. Educational meetings also facilitated the widespread implementation of testing.

The Implementation of Personalized Medicine in NSCLC in Central Europe: EGFR Testing, Histopathology, and Clinical Features (INSIGHT) observational study confirmed that EGFR mutation testing has been implemented in routine clinical practice and that patients with advanced EGFR mutation-positive NSCLC preferentially receive EGFR TKIs as first-line therapy [40]. This study in the real-world setting enrolled 1785 patients from 14 cancer centers of six Central European countries where lung cancer incidence rates are particularly high. Primary tumors were the most common sample source for mutation testing. This reflects the fact that tumor specimens were obtained by bronchoscopy or CT-guided biopsy in the majority of patients. EGFR mutation testing was done by one of the standard tests available in the respective countries. These tests included polymerase chain reaction (PCR)-RFLP, Sanger sequencing, Roche Cobas EGFR mutation test, or other methods. EGFR mutations were detected in tumors of 13.8 % of the patients. The mutation rates for histological and cytological specimens were 14.6 and 12.9 %, respectively. Exon 19 deletions were seen in 43.3 % and L858R point mutations in 28.3 % of the mutation-positive patients, respectively. T790M mutations and exon 20 insertions were seen in 1.2 and 3.6 % of the patients, respectively. Positive mutation rates varied from 10.8 to 26.7 % between centers, and this variation probably reflects differences in patient selection for EGFR mutation testing. The mutation rates were 5.7, 12.1, and 40.4 % in smokers, former smokers, and never-smokers, respectively. Data on first-line systemic therapy were available for 184 patients with advanced EGFR mutation-positive NSCLC. In 82.6 % of the patients, the mutation status was available before initiation of first-line therapy, and the majority of these patients were treated with EGFR TKIs. None of the patients without documented EGFR mutations received first-line therapy with an EGFR TKI.

The recent findings on the efficacy of third-generation EGFR TKIs in patients with T790M-positive disease require the proof of the presence of this resistance mutation in tumors at the time of disease progression in routine clinical practice. In one study on 93 patients with acquired resistance to EGFR TKI, 62 % of them had T790M mutations in their tumors at the time of re-biopsy [29]. Patients with T790M mutations had a more favorable prognosis compared to T790M-negative patients. T790M was more common in biopsies of lung/pleura tissue and lymph nodes than in more distant sites. Patients without T790M were found to more often progress in previously uninvolved organs.

Although EGFR mutation testing at the time of diagnosis has been established, several challenges remain. These include insufficient tumor tissues, intratumoral heterogeneity, heterogeneity between primary and metastatic tumors, and re-biopsy at the time of disease progression. Heterogeneity between primary tumors and lymph node metastases does occur and requires further studies in the future [41]. Several of the challenges may be overcome by liquid biopsies in the future (see below).

## EGFR monoclonal antibodies

Several anti-EGFR monoclonal antibodies have been evaluated in clinical trials (for review, see [19]). First-line chemotherapy combined with cetuximab improved outcome including overall survival in patients with advanced NSCLC [42, 43]. This benefit of cetuximab was seen in the FLEX trial, in which an enrichment strategy for EGFR expression was used for patient inclusion, and was confirmed in a meta-analysis based on 2018 patients from four randomized trials including the BMS099 trial [42, 43]. In the BMS099 trial, no significant benefit was seen when cetuximab was added to carboplatin plus paclitaxel in unselected patients [44].

Necitumumab added to first-line chemotherapy increased survival of patients with advanced squamous-cell NSCLC in the SQUIRE trial [45]. The SQUIRE trial randomized patients to chemotherapy plus cetuximab or chemotherapy alone. Chemotherapy consisted of cisplatin plus gemcitabine. The trial demonstrated increased overall survival and progression-free survival for the combined treatment. With regard to survival, the hazard ratio was 0.84 (0.74–0.96). In contrast to the SQUIRE trial, the INSPIRE trial failed to demonstrate a benefit for necitumumab added to cisplatin plus pemetrexed compared to chemotherapy alone in patients with advanced non-squamous-cell NSCLC [46].

### Predictive biomarkers

The results with regard to biomarker analysis for cetuximab and necitumumab are summarized in Table 1. Based on the results from the meta-analysis, the survival benefit of cetuximab was greatest in patients with squamous-cell carcinomas with a hazard ratio of 0.77 (95 % confidence interval (CI) 0.64–0.93) [43]. Necitumumab improved survival in squamous-cell NSCLC but not in non-squamous-cell NSCLC [45, 46].

**Table 1:** EGFR monoclonal antibodies and biomarkers in advanced NSCLC

			Overall survival hazard ratio (95 % CI)	*P*	References
Cetuximab					
FLEX	ITT	All	0.87 (0.76–0.99)	0.04	[42, 47]
		High H score	0.73 (0.58–0.93)		
	Squamous	All	0.80 (0.64–1.00)		
		High H score	0.62 (0.43–0.88)		
	Adeno	All	0.94 (0.77–1.15)		
		High H score	0.74 (0.48–1.14)		
BMS099	ITT		0.89 (0.75–1.05)	NS	[44]
Meta-analysis	ITT		0.88 (0.79–0.97)	0.009	[43]
	Adeno		0.94 (0.82–1.09)		
	Squamous		0.77 (0.64–0.93)		
	Other		0.88 (0.72–1.08)		
SWOG S0819	ITT	All	0.94 (0.84–1.06)	NS	[50]
		FISH-positive	0.83 (0.67–1.04)	0.1	
	Squamous	All	0.85 (0.67–1.08)	0.18	
		FISH-positive	0.56 (0.37–0.84)	0.006	
Necitumumab					
SQUIRE	Squamous	All	0.84 (0.74–0.96)	0.01	[45, 48]
		High H score	0.75 (0.60–0.94)		
		FISH-positive	0.70 (0.52–0.96)		
INSPIRE	Adeno	All	1.01 (0.84–1.21)	NS	[46]

High EGFR expression based on an immunohistochemistry score was shown to predict survival benefit from the addition of cetuximab to chemotherapy in the FLEX trial, whereas patients with low expression did not benefit [47]. The interaction test between EGFR expression levels of tumors and benefit from combined treatment was significant [47]. The greatest survival benefit of cetuximab with a hazard ratio of 0.62 (95 % CI 0.43–0.88) was seen in patients with squamous-cell NSCLC and high EGFR expression [47]. Similarly, high EGFR expression was associated with a greater survival benefit (hazard ratio 0.75, 95 % CI 0.60–0.94) from the addition of necitumumab to chemotherapy in the SQUIRE trial which enrolled only patients with squamous-cell NSCLC [48].

EGFR fluorescence in situ hybridization (FISH) positivity suggested benefit from cetuximab when added to first-line chemotherapy in a phase II trial [49] and led to the SWOG S0819 phase III biomarker validation study [50]. Patients were randomized into chemotherapy plus cetuximab or chemotherapy alone. Chemotherapy consisted of cisplatin plus paclitaxel with or without bevacizumab. Cetuximab did not improve outcome in the total study population. In patients with FISH-positive tumors, however, the hazard ratio for survival was 0.83 (95 % CI 0.67–1.04) in favor of chemotherapy plus cetuximab compared to chemotherapy alone. This difference was even greater in patients with squamous-cell NSCLC in whom the hazard ratio was 0.56 (0.37–0.84) for patients with FISH-positive tumors.

The benefit from necitumumab added to cisplatin plus gemcitabine in patients with squamous-cell NSCLC appeared to be greater in patients with high EGFR expression levels or those with FISH-positive tumors [48]. With regard to both parameters, however, the interaction between marker levels and clinical outcome did not reach statistical significance.

Markers other than EGFR immunohistochemistry or FISH were also evaluated as potential predictive biomarkers. KRAS mutations had no predictive value with regard to cetuximab [51, 52]. Early onset skin rash was associated with better prognosis of patients treated with chemotherapy plus cetuximab, although a predictive role could not be proven [53].

Taken together, both high EGFR expression and FISH positivity of tumors did predict benefit from EGFR-directed monoclonal antibodies when added to first-line chemotherapy of patients with advanced NSCLC.

## ALK

Echinoderm microtubule-associated protein-like 4 (EML4)-ALK fusion proteins were recently detected in 3–5 % of NSCLC, particularly in adenocarcinomas. Patients with ALK-positive tumors do benefit from treatment with crizotinib compared to chemotherapy in terms of progression-free survival and quality of life, as demonstrated for both patients pretreated with chemotherapy and chemo-naïve patients [54, 55]. Second-generation ALK inhibitors are also in development [56]. Therefore, assessment of ALK status has become diagnostic standard in routine practice for patients with advanced adenocarcinomas of the lung. Most recently, patients with ROS1-positive tumors were also shown to benefit from treatment with crizotinib [57].

### Predictive biomarkers

Techniques for assessing the ALK status are real-time RT-PCR, FISH, and direct sequencing. FISH break-apart probe remains the gold standard in the detection of ALK rearrangement in tissues. In several countries, ALK immunohistochemistry (IHC) is used as a screening test and IHC-positive cases are then confirmed by FISH.

ROS1 is also routinely assessed, mostly by means of IHC screening followed by FISH confirmation of those with positive IHC.

## Angiogenesis inhibitors

Angiogenesis inhibitors have extensively been studied in patients with advanced NSCLC. Bevacizumab added to first-line chemotherapy improved clinical outcome in patients with advanced non-squamous-cell NSCLC and has been approved for these patients [58, 59]. Nintedanib, a triple kinase inhibitors, has been studied in combination with second-line chemotherapy in patients with advanced NSCLC in the LUME Lung 1 and 2 trials. In both trials, progression-free survival was prolonged with chemotherapy plus nintedanib compared to chemotherapy alone. Nintedanib added to docetaxel also increased overall survival compared to docetaxel alone in patients with adenocarcinomas [60]. In the European Union, nintedanib has been approved in combination with docetaxel for patients with advanced adenoarcinomas who have been pretreated with chemotherapy.

### Predictive biomarkers

Translational research has focused on the characterization of predictive biomarkers for angiogenesis inhibitors. In case of bevacizumab, however, no predictive biomarker has yet been characterized for use in routine clinical practice.

In the LUME Lung 1 trial, overall survival was statistically analyzed according to a hierarchical approach under consideration of findings from the LUME Lung 2 trial which suggested benefit of nintedanib for patients who progressed within 9 months from time of initial diagnosis [60]. First, patients with adenocarcinomas and progression within 9 months from the time of initial diagnosis were analyzed. After demonstrating the benefit of combined treatment in these patients, patients with adenocarcinomas were analyzed and found to have a survival benefit from combined treatment. Finally, analysis of the total study population failed to show a significant survival benefit in these patients. These findings, first, led to the approval of nintedanib in combination with docetaxel in patients with adenocarcinomas who have been pretreated with chemotherapy and, second, also supported the predictive value of early progression.

## Immune checkpoint inhibitors

Immune checkpoint inhibitors have opened new therapeutic options in patients with advanced NSCLC. These drugs are directed against cytotoxic T lymphocyte-associated antigen 4 (CTLC4), programmed death-1 (PD-1), and PD-1 ligands PD-L1 and PD-L2. Drugs already studied or in development in lung cancer are ipilimumab, nivolumab, pembrolizumab, atezolizumab, durvalumab, and avelumab. Nivolumab and pembrolizumab have shown efficacy in phase III trials in patients with advanced NSCLC who had been pretreated with at least one line of chemotherapy [12, 13]. Response rates ranged around 20 %, and the survival gain at 1 year compared to docetaxel was about 20 % in unselected patients.

### Predictive biomarkers

Research has focused on the characterization of those patients who derive the benefit from immune checkpoint inhibitors. Over-expression of PD-L1 or PD-L2 on tumor cells has been explored as a potential predictive biomarker. In case of nivolumab, high expression of PD-L1 was associated with greater benefit from nivolumab in patients with non-squamous-cell NSCLC, whereas no such difference in outcome was shown for patients with squamous-cell NSCLC [13, 14]. In the atezolizumab randomized phase II trial in pretreated patients with advanced NSCLC, PD-L1 expression was evaluated by immunohistochemistry by means of the SP142 antibody on tumor samples and tumor-infiltrating immune cells [61]. The greatest improvement appeared to occur in patients with the highest PD-L1 levels on tumor cells or on immune cells.

Other studies evaluated tumor-infiltrating immune cells, interferon-gamma, and other factors of the tumor microenvironment. Gene analyses focusing on mutation loads and mismatch repair deficiency are also promising. NSCLC and melanomas, for which immune checkpoint inhibitors are already in clinical use, have the highest mutation rates among all malignant diseases [5]. These mutations result in high levels of mutated proteins which will then serve as tumor antigens and may make these cancers susceptible for immunotherapy.

Standardization of testing remains a major challenge for the clinical development of predictive biomarkers. Differences in type of markers, test methods, and cutoff levels between trials make comparisons of results between trials very difficult.

Overall, the identification of predictive biomarkers for immune checkpoint inhibitors is complex. Development of reliable predictive biomarkers has to consider tumor heterogeneity, differences between primary tumors and metastases, dynamic changes of biomarkers over time, test methods, and other factors. With regard to testing, tumor volume and tumor cell density are important. Based on this complexity, no predictive biomarker has yet been characterized for use in routine clinical practice.

## Challenges of molecular testing in routine clinical practice

In order to establish and implement molecular testing in routine practice, co-operation and communication between the treating physicians and the pathologists are of particular importance as stressed in the First European Workshop on EGFR mutation testing [25]. The detailed logistics of testing, however, are best established at the local level because strategies that work in one cancer center may not necessarily be the most appropriate ones in others. Although the decision which patients should be tested is the primary responsibility of the treating physician, this strategy can be time consuming and may delay the time when test results will be available for the clinician. Therefore, many cancer centers have adopted a reflex testing strategy where the pathologists forward the tumor samples for molecular analysis as soon as they have established the histological diagnosis. This strategy is more efficient and allows test results to become available for the treating physician within clinically acceptable time periods.

### Tumor specimens

Tumor specimens obtained at the time of initial diagnosis are also used for molecular testing in routine clinical practice. The selection of the tumor site for biopsy depends on its accessibility and must consider being associated with the lowest risk and burden for the patient. Modern biopsy techniques often provide only small tumor specimens, and this may impact on molecular analysis because histological confirmation of cancer remains the most important goal of tumor biopsies. Only after the diagnosis has clearly been established, the remaining tissue can then be used for molecular analysis. This strategy may result in insufficient tumor tissue for molecular analysis and may require re-biopsy, dependent on the clinical situation. Therefore, methods for tumor sampling need improvement in order to provide tissue that will be sufficient for both histological and molecular diagnosis. In the future, liquid biopsies will become an important additional source for molecular analysis.

Source, handling, and fixation of tumor specimens have to be standardized. Pathologists should provide physicians who will perform the biopsy with a standardized protocol on sampling and handling of tumors. Based on the INSIGHT study, tumor samples were obtained by bronchoscopy, EBUS/EUS, and computer-tomography-guided biopsy or surgery [40]. Primary tumors, lymph nodes metastases, and distant metastases were used for pathological and molecular diagnosis in 85.7, 2.6, and 11.7 % of the patients, respectively. Pathological diagnosis was based on histology (with or without cytology) in 82.2 % and on cytology alone in 17.8 % of the patients.

### Re-biopsies

Molecular analysis is increasingly becoming important also at the time of tumor progression. As an example, the clinical prescription of third-generation EGFR TKIs requires the proof of the presence of the T790M resistance mutation in tumor cells. With regard to the site of re-biopsy, the most easily accessible tumor site should be selected in routine clinical practice. Re-biopsy at the time of tumor progression can be particularly challenging because patients may have poor performance status, may suffer from heavy symptom load, or may refuse re-biopsy. Therefore, non-invasive approaches such as liquid biopsies may become promising sources for tumor genotyping.

### Liquid biopsies

The procedure of taking blood samples to detect tumor-specific genetic alterations is termed “liquid biopsy.” Liquid biopsies are currently studied as a potential alternative source for molecular analyses. Tumor materials obtained from the peripheral blood include circulating cell-free DNA (cfDNA), circulating tumor cells (CTCs), and exosomes. cfDNA is composed of small fragments of nucleic acids that are not associated with cells or cell fragments, whereas circulating tumor cells represent intact, viable cells that can be purified from blood. Exosomes are extracellular vesicles that contain RNA, DNA fragments, proteins, and metabolites.

Blood samples can easily and repeatedly be taken and may also allow detecting genetic heterogeneity of tumors. Blood-based analytic approaches may allow real-time monitoring of the total tumor burden and the detection of mutations during treatment.

#### Circulating cell-free DNA

Circulating cell-free DNA (cfDNA) consists of DNA fragments which originate from tumor cells but also normal cells that are released into the bloodstream. Although cancer patients tend to have higher levels of cfDNA, the levels of tumor-derived, circulating cfDNA are usually low. Therefore, the discrimination of mutated cfDNA from wild-type cfDNA requires high sensitivity of the test methods.

The following methods for the analysis of tumor-derived cfDNA are available: digital polymerase chain reaction (PCR); beads, emulsion, amplification, and magnetics (BEAMing); pyrophosphorolysis-activated polymerization (PAP); and tagged-amplicon deep sequencing (TAM-Seq). The sensitivity of these techniques is limited due to low number of tumor-derived cfDNA copies and error rates of the polymerases used for PCR. Digital platforms are more sensitive than non-digital platforms as recently demonstrated for the detection of *EGFR* mutations in circulating cfDNA of patients with advanced NSCLC [62].

Liquid biopsies were used to detect and monitor mutations [39, 62–65]. In a recent study, next-generation sequencing of cell-free plasma DNA (cfDNA) from patients with advanced lung cancer whose tumors had developed resistance to osimertinib revealed an acquired EGFR C797S mutation [39]. Another study demonstrated the feasibility of detecting and monitoring of EGFR mutations in cell-free plasma DNA from patients with EGFR-mutated advanced NSCLC undergoing treatment with erlotinib [64]. Serial quantifications of cell-free plasma DNA demonstrated that the T790M mutation could be detected several weeks before radiographic progression.

In an exploratory study of 40 patients with EGFR mutation-positive tumors progressing on EGFR TKI therapy, the T790M genotype from tumor biopsies was compared with findings from simultaneously collected circulating tumor cells and circulating tumor DNA [65]. T790M genotypes were successfully obtained in 75 % of the tumor biopsies, 70 % of the circulating tumor cell samples, and 80 % of circulating tumor DNA samples. T790M was detected in 47–50 % of patients with a concordance rate of 57–74 %. Circulating tumor cell–based and circulating tumor DNA–based genotyping were each unsuccessful in 20–30 %. The combination of both tests, however, allowed successful genotyping in all patients. T790M mutations were detected in 35 % of the patients in whom tumor biopsy was negative or indeterminate. Therefore, tumor biopsy and blood-based analyses may give discordant results, but the use of complementary approaches may provide more relevant results.

Liquid biopsies will contribute to a deeper insight in the molecular development of resistance mechanisms and will enable continuous monitoring of the tumor genotype including the detection of molecular relapses prior to radiographic or clinical progression. The results of liquid biopsies will have to be compared with those from tumor biopsies and will have to be shown that they allow clinically reliable treatment guidance. Therefore, further research is necessary before liquid biopsies can be recommended as a routine method for guiding treatment of patients with molecular alterations in their tumors.

## Abbreviations

ALK: Anaplastic lymphoma kinase
EGFR: Epidermal growth factor receptor
EML4: Echinoderm microtubule-associated protein-like 4
FISH: Fluorescence in situ hybridization
IHC: Immunohistochemistry
NSCLC: Non-small-cell lung cancer
PCR: Polymerase chain reaction
SCLC: Small-cell lung cancer
TKI(s): Tyrosine kinase inhibitor(s)
